# Release of the National Scheme’s Aboriginal and Torres Strait Islander Health and Cultural Safety Strategy 2020-2025; the impacts for podiatry in Australia: a commentary

**DOI:** 10.1186/s13047-021-00466-8

**Published:** 2021-05-10

**Authors:** James M. Gerrard, Shirley Godwin, Vivienne Chuter, Shannon E. Munteanu, Matthew West, Fiona Hawke

**Affiliations:** 1 College of Health, Medicine and Wellbeing, School of Health Sciences, University of Newcastle, Ourimbah, NSW 2258 Australia; 2 Discipline of Podiatry, School of Allied Health, Human Services and Sport, La Trobe University, Melbourne, 3086 Australia; 3 Rural Department of Nursing & Midwifery, La Trobe Rural Health School, La Trobe University, PO Box 199, Bendigo, 3552 Australia; 4 Priority Research Centre for Physical Activity and Nutrition, University of Newcastle, Ourimbah, NSW 2258 Australia; 5 La Trobe Sport and Exercise Medicine Research Centre, School of Allied Health, Human Services and Sport, La Trobe University, Melbourne, 3086 Australia

**Keywords:** Cultural safety, Aboriginal and Torres Strait Islander, First Nations, Indigenous, Podiatry

## Abstract

**Background:**

Developing since colonisation, Australia’s healthcare system has dismissed an ongoing and successful First Nations health paradigm in place for 60,000 years. From Captain James Cook documenting ‘very old’ First Nations Peoples being ‘far more happier than we Europeans’ and Governor Arthur Phillip naming Manly in admiration of the physical health of Gadigal men of the Eora Nation, to anthropologist Daisy Bates’ observation of First Nations Peoples living ‘into their eighties’ and having a higher life expectancy than Europeans; our healthcare system’s shameful cultural safety deficit has allowed for an Aboriginal and Torres Strait Islander child born in Australia today to expect to live 9 years less than a non-Indigenous child. Disproportionately negative healthcare outcomes including early onset diabetes-related foot disease and high rates of lower limb amputation in Aboriginal and Torres Strait Islander Peoples contribute to this gross inequity.

**Main body:**

In 2020, the Australian Health Practitioner Regulation Authority released the National Scheme’s Aboriginal and Torres Strait Islander Health and Cultural Safety Strategy 2020–2025 - empowering all registered health practitioners within Australia to provide health care to Aboriginal and Torres Strait Islander Peoples that is inclusive, respectful and safe, as judged by the recipient of care. This recently released strategy is critically important to the podiatry profession in Australia. As clinicians, researchers and educators we have a collective responsibility to engage with this strategy of cultural safety. This commentary defines cultural safety for podiatry and outlines the components of the strategy in the context of our profession. Discussion considers the impact of the strategy on podiatry. It identifies mechanisms for podiatrists in all settings to facilitate safer practice, thereby advancing healthcare to produce more equitable outcomes.

**Conclusion:**

Aboriginal and Torres Strait Islander Peoples access health services more frequently and have better health outcomes where provision of care is culturally safe. By engaging with the National Scheme’s Aboriginal and Torres Strait Islander Health and Cultural Safety Strategy, all registered podiatrists in Australia can contribute to achieving equity in health outcomes for Aboriginal and Torres Strait Islander Peoples.

## Note

This work includes the nomenclature; Aboriginal and Torres Strait Islander Peoples, First Nations Peoples and Indigenous. Neither singularly, nor collectively do they adequately represent the immense diversity of language groups and cultural values across this continent’s Traditional Custodians and rightful owners [1, 2]. The terms decolonise, decolonisation and decolonising methodology throughout this work describe being inclusive of Aboriginal and Torres Strait Islander worldview and holistic conceptualisation of health and well-being [3].

## Background

Since colonisation, Australia’s healthcare system has been dismissive of an ongoing and successful First Nations health paradigm in place for more than 60,000 years. Historical and current deficits in the modern healthcare system have culminated in Aboriginal and Torres Strait Islander Peoples experiencing a burden of disease that is 2.3 times the rate of non-Indigenous Australians [4], where burden of disease measures the impact of living with illness and injury and dying prematurely [4]. The cultural safety deficit in our healthcare system impacts all First Nations Peoples, and all areas of health. For the lower limbs, Aboriginal and Torres Strait Islander Peoples have an increased likelihood of experiencing diabetes related foot complications compared to non-Indigenous Australians [5]. In fact, a state-wide audit of all amputations performed in Western Australia for years 2000 to 2008 found among people 25 to 49 years of age with diabetes, major amputations were 38 times more likely, and minor amputations 27 times more likely in Aboriginal and Torres Strait Islander Peoples than in non-Indigenous Australians. In that audit, 98% of amputations in Aboriginal and Torres Strait Islander Peoples were associated with diabetes [6]. These data are especially concerning given that the 5-year mortality rate following lower limb amputation is 57% [7] and highlight that a podiatrist working within the Australian healthcare system must be able to provide culturally safe health care to First Nations Peoples.

In 2020, the Australian Health Practitioner Regulation Agency (AHPRA) and the National Boards released The National Scheme’s Aboriginal and Torres Strait Islander Health and Cultural Safety Strategy 2020–2025 with an aim to produce consistency and quality improvement in matters of Aboriginal and Torres Strait Islander health and cultural safety across the National Scheme. The strategy is the first national guideline shaping podiatry and other professions’ cultural practice, recognising that cultural safety is a critical component of patient safety. This commentary provides an overview of cultural safety in podiatry and discusses how the National Scheme’s Aboriginal and Torres Strait Islander Health and Cultural Safety Strategy 2020–2025 impacts our profession, as individual practitioners and researchers, and as members of healthcare organisations, higher education institutions, and boards of governance.

## Main text

### Is podiatry culturally safe?

Podiatry is a knowledgeable and progressive allied health profession. It is a field that involves protecting quality of life. As podiatrists we keep people comfortable, mobile and active, we perform small surgical procedures as part of common practice, and we manage wounds to save limbs from amputation. Podiatry nurtures childhood development and cares for the elderly. Our progressive discipline provides a pathway to surgery and endorsed scheduled medicines prescribing rights. Podiatry produces robust research underpinning evidence-based practice. Our profession is full of dedicated hard-working, caring practitioners implementing patient-centred care in the compassionate pursuit of optimising health outcomes in its patients. Podiatry has strong governance through the National Registration Board; The Podiatry Board of Australia [8].

Reading this as podiatrists trained in a Western model of healthcare we are likely to interpret the above as; *knowledgeable* in terms of Western science; *elderly* as non-Indigenous octogenarians supported by an array of social systems designed specifically to support their long life expectancy; *research* that doesn’t include Aboriginal and Torres Strait Islander perspectives nor their Peoples as participants; *health outcomes* being directed by biomedical markers rather than spiritual or cultural elements, or connection to Country; and *governance* without consideration of treaty. So, is podiatry culturally safe?

This calls all registered podiatrists to consider whether we, individually and collectively, are engaged in culturally safe care.

### What is cultural safety?

The National Scheme’s Aboriginal and Torres Strait Islander Health and Cultural Safety Strategy 2020–2025 released through AHPRA defines cultural safety today as ‘the ongoing critical reflection of health practitioner knowledge, skills, attitudes, practising behaviours and power differentials in delivering safe, accessible and responsive healthcare free of racism’ [9]. It is judged by the recipient of care, Aboriginal and Torres Strait Islander Peoples [9].

Cultural safety is a concept developed by Dr. Irihapeti Ramsden, Ngāi Tahu/Rangitāne, [10, 11], a Māori nurse and nursing educationalist [12]. Dr. Irihapeti Ramsden’s work questions power relations between health practitioners and recipients of care, focusing on practitioner approach, sentiments, behaviour, biases and worldview [13]. Becoming ever more prominent in health service delivery [10, 11], Ramsden’s cultural safety framework addresses colonised health care methods and their impacts by providing a lens for health practitioners to gaze inwardly at themselves, whilst establishing ‘a mechanism which allows the recipient of care to say whether or not the service is safe for them to approach and use’ [13].

### Why is cultural safety important in podiatry?

Whilst AHPRA currently mandates culturally safe practice as a requirement for registration for some health professionals [14], the recent release of the National Scheme’s Aboriginal and Torres Strait Islander Health and Cultural Safety Strategy 2020–2025 targets health inequity experienced by Aboriginal and Torres Strait Islander Peoples through nationally consistent standards, codes and guidelines across *all* practitioner groups within the National Scheme [9].

### We all need to understand the National Scheme’s Aboriginal and Torres Strait Islander health and cultural safety strategy 2020–2025

The Aboriginal and Torres Strait Islander led Health and Cultural Safety Strategy released in 2020 is a consolidated, articulated strategy aimed at achieving Aboriginal and Torres Strait Islander health equity [9]. Acknowledging Aboriginal and Torres Strait Islander Peoples never ceded sovereignty [9], and the past and current impacts colonisation and racism have on Aboriginal and Torres Strait Islander health and well-being [9], the strategy outlines key factors in developing cultural safety (Table 1).

**Table 1:** Four key elements to ensure culturally safe and respectful practice; adapted from the National Scheme’s Aboriginal and Torres Strait Islander Health and Cultural Safety Strategy 2020–2025 [9]

Four key elements for us to ensure cultural safety	
1	Acknowledge colonisation, systemic racism and social, cultural, behavioural and economic factors which impact individual and community health
2	Acknowledge and address individual racism, our own biases, assumptions, stereotypes and prejudices and provide care that is holistic, free of bias and racism
3	Recognise the importance of self-determined decision-making, partnership and collaboration in healthcare which is driven by the individual, family and community
4	Foster a safe working environment through leadership to support the rights and dignity of Aboriginal and Torres Strait Islander Peoples and colleagues

The strategy reinforces that, to provide culturally safe podiatry care, we need to provide treatment deemed safe by the recipient of care. It also reinforces that Western biomedical practice is founded upon knowledge and views that differ from Aboriginal and Torres Strait Islander considerations of the physical, psychological and spiritual facets of holistic well-being and of kinship and connectedness to Country [15–17].

We need to be aware that individual clinicians, health organisation governance, researchers and tertiary sector education providers, can all (even unknowingly) perpetuate Aboriginal and Torres Strait Islander healthcare disparities through attitudes and practices [18]. We need to realise, too, that cultural safety is not achieved in a one-off ‘tick box’ workshop, but is developed through progressive, inclusive, systemic and systematic change. This strategy provides mechanisms, a collection of resources working together, ‘to change minimum levels of practice that registered health practitioners must meet, as well as the standards for the educational courses that lead to registration, the vast reach of the National Scheme puts its entities in a unique position to affect real change to patients and communities’ [9].

### We all need ongoing cultural self-awareness and self-reflection

Dr. Ramsden’s cultural safety framework establishes a career-long journey [19], developing our realisation that one’s own culture may disadvantage recipients of care [20]. Initially, we need to develop our cultural self-awareness. Cultural self-awareness aids critical self-reflection and exploration of our positioning, assumptions, biases and pre-conceptions relating to cultural elements such as appearance, ethnicity, politics, religion, age, and language for example, which all underlie practice [20]. An inward gaze shifts our cultural underpinning into critical focus for reflection [15]. This gives recognition to the varying socio-economic and spiritual context in which people exist, facilitating our understanding of difference [13, 19]. Secondly, we validate our differences when we self-explore our own worldview and the impacts this has upon our behaviours and practice [13, 17, 19]. Finally, resulting achievement of our changed practice culminates in safe caregiving as defined by the recipient of care [13, 19]. This process then repeats ongoingly.

As podiatrists engaged in a continual cultural self-awareness process, we may better consider how our own culture and its unique differences can influence the way we deliver health care and how it is experienced. A starting point in this process is our recognition of diversity. To treat everyone in our care the same, regardless of difference, may be a commonly held view of many of us, mistakenly conceptualising this as caregiving free of disadvantage. This approach of neutrality, however, undermines equity in health care. Any view of podiatry provision that is not fully understanding of differences in health status between population groups that are systematic in their unequal distribution, socially produced, needless and discriminatory [21] actually erodes cultural safety.

Self and critical reflection is a mandated component of our professional development [22]. Reflective practice explores experience, focusing on emotions, decision making, beliefs and behaviours that can be modified to develop knowledge and progress ability. Self-reflecting upon our attitudes and practices can create an inclusive and safer environment for Aboriginal and Torres Strait Islander Peoples accessing healthcare [23] and create positive experiences within the healthcare system.

### We need to apply the principles of cultural safety

To apply the principles of cultural safety to our podiatry practice in Australia [19] we need to instil Aboriginal and Torres Strait Islander presence, empowerment and expertise in initiatives and approaches to Aboriginal and Torres Strait Islander health issues [24]. By reflecting on our own practice; seeking to minimise power differentials; engaging in discourse with the patient; ensuring that we do not diminish, demean or disempower through our actions; and undertaking the process of decolonisation [19, 25], we can continuously modify purely Western biomedical podiatric approaches to holistic and culturally safe caregiving [24]. A process of decolonisation in podiatry practice goes beyond declarative imagery and print in workplace settings. It has us actively and progressively rebutting the colonising monopolisation Western knowledge systems impart on theory and practice [26] through our actions and behaviours. Actions might include demonstrating understanding of connection to Country by discussing where people are from and how Country facilitates sense of belonging and well-being. Behaviours such as prolonged direct eye contact for example, may change to curb our own cultural influences on care provision. We might actively listen to, and learn from, patients expressing their own cultural belief so as it may be privileged in their management and drive self-determination of health care. We might advocate for changes in policies resulting in respectful relocation of treatment settings, longer or flexible appointment times, and communication methods inclusive of family, Elders and Aboriginal and Torres Strait Islander health workers. Our decolonisation of practice might have us engage in lifelong cultural safety learning, so that we continuously counter inadvertent misrepresentation and dehumanising of Aboriginal and Torres Strait Islander Peoples [27].

### We should develop cultural capability in our profession

Culturally capable and safe learning and practice provides realistic purpose in the pursuit of health equity [28, 29] - where capability implies a long, developing learning and ever-responsive behaviour [29], and safety promotes self-reflection and learning with, and from, Aboriginal and Torres Strait Islander Peoples. Podiatry today needs to move on from competency-based cultural development paradigms that epitomise acquiring knowledge about a culture from an anthropological view. This is not a change in learning terminology, but a change in learning method. Achieving competence can be deemed to establish expertise in cross-cultural knowledge which reinforces power imbalance [28], deflects clinician considerations of power, privilege and biases [28], alienates or ‘others’ those not belonging to dominant cultures [28], homogenises Aboriginal and Torres Strait Islander Peoples [30], reinforces simplistic cultural stereotypes [30], and victim-blames poor health outcomes on the affected cultures, perpetuating deficit discourse [31].

Based on this, competency-based learning methods apply a defined and very limited set of learned outcomes against diverse and layered cultural make-up; inadequate and inappropriate for developing principles and practical steps to facilitate equity in health care provision by healthcare organisations and healthcare workforce [28]. Noted is the lack of evidence for “culturally competent” healthcare professionals providing positive health outcomes for Aboriginal and Torres Strait Islander patients [32]. As a profession today, learning to become progressively more culturally capable and safe, better places us to achieve health equity as it involves continual self-reflection and ongoing addressing of power differentials subsequent to colonial history [28].

### We must minimise implicit bias and stereotypes

Generalisations can be the beginning of learning about another’s culture, however, without developing a deeper understanding of cultural capability and safety, preconceptions and misinformation can lead to the formation of stereotypes, ongoing stereotyping, prejudice and discrimination [33].From the time of colonisation, Aboriginal and Torres Strait Islander Peoples have been subjected to generalisations based on; lack of understanding of heterogeneity of cultures; equating difference with inferiority; and stereotypes based on misinformation and bias [33].

Bias is inherent to mental processing and although it can be advantageous in speeding up decision making and motivating problem-solving [34], its influence on our thought processes creates vulnerability to false assumptions and reasoning flaws [35]. Cognitive bias is systematic error in thinking that influences decisions we make [36], for example, giving weight to information because we heard it first. Confirmation bias is the interpreting of evidence in ways that tend to affirm existing beliefs, theories or expectations [37]. These biases may be conscious or explicit, but they may also be implicit. Implicit bias is unintended or unconscious, with studies demonstrating that implicit bias can cloud clinical decision-making [38, 39]. An example of this is clinicians implicitly linking treatment adherence with being white/Caucasian [38].

It is important for us to understand that developing implicit biases can proceed in a gradual, subtle way and can manifest generalised and often negative ethnocentric stereotypical views about a particular group [33]. In 2014, research data from an Australian Public Health Advocacy Institute media project revealed 74% of the media coverage portraying First Nations health issues to be negative [40]. Such negative narrative perpetuates unconscious biases among the wider population [40], a notion supported by recent research demonstrating that the majority of Australians sampled, regardless of background, held an implicit or unconscious bias against Aboriginal and Torres Strait Islander Peoples [41]. Translated across the social, economic and healthcare spheres this impacts heavily on the health and well-being of First Nations Peoples [40].

Changed environments of organisations and individuals of good intention are not always adequate in reducing implicit bias and stereotyping and their impacts [39]. Effective mechanisms for reducing implicit bias include stereotype replacement and perspective taking (being aware of more than our own feelings, motivation and worldview) [39], and preventing biased decision-making through awareness of unconscious thought processes and improved decision-making conditions [39].

In order to deliver culturally safe podiatry practice we need to understand bias, recognise biases within ourselves through careful self-reflection, and provide care free from any prejudicial implicit bias [9].

### We need to know Australian history and recognise the ongoing impacts

Understanding the history of the continent now known as Australia is an important first step in developing culturally safe podiatry practice. The racist systems, laws and processes this country was founded upon positioned one culture as inferior. This continues to be a powerful influence on health and well-being for Aboriginal and Torres Strait Islander Peoples today. Knowledge of this history allows for not only a greater understanding of current health challenges, but also a greater appreciation of the resistance of oppression [42] and cultural strength of First Nations Peoples. As the world’s oldest living cultural and political societies [43], Aboriginal and Torres Strait Islander Peoples have lived in this land in excess of 60,000 years [44]. Prior to colonisation there were many unique kinships and cultural boundaries, as well as shared spiritual beliefs and laws throughout Australia [45], spread across over 500 First Nations [45] and approximately 260 language groups [44].

Aboriginal and Torres Strait Islander Peoples and their connection with the land was not recognised by the early colonists, nor their successors; with their traditional and successful scientific, scholarly, social, health, agricultural and governance systems remaining largely unrecognised. Australia was founded upon the notion of *terra nullius*, legally deemed as unoccupied or uninhabited, and not until 1992 did the High Court of Australia reject *terra nullius* as a legitimate source of legal foundation [46]. Colonising nineteenth century policies of ‘*protection*’ restricted Aboriginal and Torres Strait Islander Peoples from towns, biased judicial powers and increased imprisonment rates [47–49]. The 1901 Commonwealth Constitution instated seven decades of ‘White Australia policy’ promoting and maintaining racial and cultural homogeneity [47–49], with Aboriginal and Torres Strait Islander Peoples excluded from census and from lawmaking process until the Constitution Alteration (Aboriginals) 1967 (Act No 55 of 1967) [50]. Concurrent twentieth century policies of ‘assimilation’ inflicting ‘intergenerational trauma’ [51] by forcibly removing up to 1 in 3 Aboriginal and Torres Strait Islander children from their families between 1788 and 1901 [52] and between 1910 and 1970 [47–49, 53, 54] leaves the legacy of colonisation as the breaking down of Aboriginal and Torres Strait Islander cultural continuity, connection to Country, laws, language, families, ceremonies, economic independence and kinship systems. All things linked to health and well-being [27, 55] and that give identities, empowerment and humanity.

Australian history to this point has culminated in First Nations Peoples experiencing a burden of disease that is 2.3 times the rate of non-Indigenous Australians [4], where burden of disease measures the impact of living with illness and injury and dying prematurely [4]. Personal histories, post-colonial legacies and the cultural perceptions of Aboriginal and Torres Strait Islander Peoples can bewilder health professionals if they have inadequate knowledge of these important issues. Poor understanding of the past and ongoing injustices undermines respect, devalues integrity and compounds communication barriers. It can therefore have a devastating impact on health outcomes for First Nations Peoples [56].

An understanding of historical determinants of health, including the notion of Country as a protective factor, is key to providing culturally safe podiatry care to Aboriginal and Torres Strait Islander Peoples [27]. Without a knowledge of Australian history and its ongoing impacts, a non-Indigenous Australian podiatrist can have a sense of inadequacy and incapability.

### We must truly understand racism and its impact upon the health and well-being of First Nations Peoples

The National Aboriginal and Torres Strait Health Plan 2013–2023 aims for all health care to be free of racism [16], and the National Scheme’s Aboriginal and Torres Strait Islander Health and Cultural Safety Strategy 2020–2025 is committed to eradicating racism from the healthcare system [9]. Racism combines prejudicial beliefs, emotions, and discriminatory behaviours and practices toward people of a given cultural group [57]. It is a social construct founded upon biological characteristics such as skin tone and facial features [58] which creates avoidable and unfair inequity [59] and has no scientific basis [58]. Yet, it is the use of the notions of power relations used to identify different cultural groups as inferior and superior which enabled the colonisation of Australia.

Racism is a key social determinant of health for Aboriginal and Torres Strait Islander Peoples [16, 60]. It causes psychological distress, negative self-esteem and maladaptive responses, all of which reduce health and well-being [61]. Overt and covert racism, prejudice and discrimination reduce Aboriginal and Torres Strait Islander access to, and quality of, health care [62]. This leads directly to physical, biological and mental health disadvantage [62]. Australian research shows Aboriginal and Torres Strait Islander adults are four times more likely to experience racism over a one-year period than their non-Indigenous counterparts [60], with 97% of Aboriginal and Torres Strait Islander Peoples reporting at least one racist incident over that period [63]. Shamefully, in a setting that should be nothing less than completely inclusive, approximately one third of racist incidents reported in research cross-sectional surveys occurred within healthcare settings [63]. Reported incidents included name calling, being treated as less intelligent, and being sworn at or ignored, and, when occurring in the healthcare setting, have an association with increased psychological distress over and above what would be expected elsewhere [63].

In addition to interpersonal racism, numerous studies propose systemic or institutional practices impact on delivery of health care, with Aboriginal and Torres Strait Islander Peoples being less likely to receive treatment than non-Indigenous people with the same health need requirements [15, 61, 62]. Institutional racism manifests within our healthcare system [64] where white is the unseen ‘normal’ that is rarely interrogated, and against which minority groups of people are positioned [18]. Such racism is expressed through economic and political systems, higher education, and health policy. Maintained by lack of critical appraisal of frameworks within the context of culture, institutional racism perpetuates Aboriginal and Torres Strait Islander health care disparities [18] by failing to provide appropriate service to people based on racial or cultural difference.

A multi-tiered approach is required to identify and reduce racism in Australian healthcare [18, 65, 66], implemented via an individual cognitive and interpersonal level, an organisational policy level and at a government level, by political will [65]. As individuals, we need to understand the false dichotomy of an over-simplified good/bad racist binary [67], recognising that even well-intentioned care givers, teachers and researchers may unconsciously and unsuspectingly perpetuate racist institutional policy and practice they are trying to confront and dismantle. There is great need for us to refine perceptions and control behaviours at discovery of any racist construct or role in racist systems. To be able to self-examine, we need to welcome feelings of uneasiness and limit reactionary defensiveness to quell stress in response to race-related issues [67]. Key to the process of repairing the damage of racism is us being able to recognise racism, collectively discuss it, educate each other about it, and work together towards eliminating it from podiatry.

In structuring organisational governance, evidence identifying racism as both a barrier to accessing care and to receiving indicated interventions [68] needs to be utilised to underpin individual and institutional cultural safety. There is evidence to support the use of the following to overcome racism and improve the cultural safety of services: culturally appropriate tools within hospital education, inclusion of families, culturally appropriate warning information [69], cultural safety learning, health worker toolkits, and partnerships with mentors from Aboriginal and Torres Strait Islander organisations [15, 70]. As organisations, a model of healthcare inclusive of robust critical reflection directing service provision needs to drive policies whilst disintegrating racist premises [18].

The actions and commitment of our practitioners, accreditors, educators, researchers and policy makers [15] are a means to eradicate racism at personal and institutional levels, and to establish trust in all podiatry settings.

### We must acknowledge white privilege

White privilege is a system of advantage that is available to white people and unavailable to others. It is inextricably linked to racism, biases and stereotyping at both individual and institutional levels [71, 72]. White privilege is a discriminator, impacting preventive care [72] and fundamentally fuelling the disparity in the mortality rate between First Nations Peoples and non-Indigenous Australians. Where white people learn about racism as a construct that inflicts disadvantage, its consequence, white privilege, is not often considered as providing advantage [73].

To address this, it is incumbent upon us as health professionals to acknowledge the systemic white privileges produced through colonisation and the pursuant postcolonial advantages afforded to white Australian society [29, 74]. Additionally, we need to reflect upon our own positioning in relation to white privilege [29] to unpack the realisation that working from a base of unacknowledged white privilege can maintain oppression [73]. For us to develop a decolonised podiatry model, we need to engage the mechanisms of acknowledging privilege and deconstructing power differentials favouring a ‘white normal’ [29]. This can be achieved through self-reflection to produce personal change and development, and by using positions in governance to re-write policy or to reconfigure power systems across a broader base [73] that includes Aboriginal and Torres Strait Islander ways of being, ways of knowing and ways of doing.

### We need to change the conversation

A further mechanism for enacting culturally safe practice is to change the conversation from a deficit discourse to a strength-based narrative. Deficit discourse positions Aboriginal and Torres Strait Islander identity negatively [75]. It intersects with health and education [76] and conjures the perception that Aboriginal and Torres Strait Islander Peoples themselves are a failure or inferior, and are responsible for problems such as their health outcomes disadvantage [31]. Deficit metrics such as the Australian Government’s ‘Closing the Gap’ initiative can support such discourse, reducing Aboriginal and Torres Strait Islander Peoples to a single entity defined as unable to achieve a benchmarked normality represented by all non-Indigenous populations [76]. They can also drive the perception for podiatrists of overwhelming and insurmountable problems. Deficit discourse simply does not consider causes of inequity [31], and it ignores the strength, diversity, resilience and pride of Aboriginal and Torres Strait Islander Peoples. There is significant reason to believe that media and political deficit discourse saturation in this country has a substantial influence upon First Nations Peoples [75], with evidence of impact upon health and well-being outcomes [27, 76].

Strength-based discourse incorporates concepts of resilience, protective factors (such as Country) and decolonisation methodology [76]. Incorporating First Nations conceptualisations of strength-based approaches encapsulating resistance to oppression is fundamental to changing the conversation [42]. This hears Indigenous voice and shifts power imbalance whilst educating [42]. The National Scheme’s Aboriginal and Torres Strait Islander Health and Cultural Safety Strategy 2020–2025 exemplifies strength-based discussion, acknowledging firstly that Aboriginal and Torres Strait Islanders are ‘the beneficiaries of 60,000 years of science, knowledge and paradigms that can inform better health care for all Australians’ [9] and secondly ‘that healthcare science has been practised by Aboriginal and Torres Strait Islander Peoples for millennia on this continent, and that Western healthcare and science has a lot to learn from the original human healers’ [9]. Strength-based emphasis of health promoting factors re-focuses upon progress and achievement to generate positive public perceptions and high self-esteem, and facilitates pride and progress [77]. Literature suggests that Elders’ voices, along with a strength-based approach inclusive of genuine relationships, critical reflection upon Australia’s political, social and historical contexts, and process and outcome evaluation [78] could be used to progress culturally capable and safe well-being services, continuing professional development delivery and health degree teaching [78].

### We must listen to Aboriginal and Torres Strait Islander voices

The Uluru statement calls for a First Nations voice enshrined in the Constitution; for Aboriginal and Torres Strait Islander Peoples to be heard [79]. Listening to Elders is perhaps the most important mechanism in developing capable and safe provision of care. As tales of stereotyped identity [80] are common sources from which non-Indigenous Australians draw information about Aboriginal and Torres Strait Islander Peoples [81], individuals, organisations and education providers need to be prepared to put aside preconceived ideas, challenge common misconceptions of First Nations Peoples, and truly listen to what Aboriginal and Torres Strait Islander Peoples have to say. According to Marcia Langton, Chair of Australian Indigenous Studies, University of Melbourne, ‘most Australians do not know how to relate to Aboriginal people. They relate to stories told by former colonists’ [82]. Listening to, hearing, privileging and valuing Aboriginal and Torres Strait Islander voices supports truth-telling. Professor Megan Davis, Associate Professor Rosalind Dixon, Associate Professor Gabrielle Appleby and Noel Pearson tell us in an edited transcript of The Uluru Statement, ‘truth-telling opens the way for justice, healing, the restoration of dignity and on those bases, reconciliation’ [79]. Truth-telling validates Aboriginal and Torres Strait Islander perspectives and acknowledges the lived realities of Aboriginal and Torres Strait Islander Peoples - elements integral to Aboriginal and Torres Strait Islander health and healing.

Listening to Aboriginal and Torres Strait Islander voices is collaborative. It challenges and changes practices, and culturally co-creates a decolonised space of new knowledge, insight and understanding [80, 83]. A core performance indicator of culturally safe frameworks [84] stipulated for all levels of healthcare design and delivery [84] is the inclusion of local Aboriginal and Torres Strait Islander voices. This empowers the experts in Aboriginal and Torres Strait Islander health, Aboriginal and Torres Strait Islander Peoples themselves, to implement, direct and evaluate culturally safe initiatives [24].

### We must implore cultural humility

Cultural humility is the awareness of the limited extent to which we can meaningfully understand someone else’s culture. It is a humble and respectful approach towards people of differing cultures involving continual self-evaluation and critique to recognise our own cultural biases [85]. Instilling mutual empowerment and respect between podiatrist and patient [86] and involving advocacy partnerships with communities [85], cultural humility requires recognising the patient as the expert in their own culture and cultural aspects of their health and well-being, and we, the podiatrists, as the learners. In egoless and supportive interactions [86], cultural humility highlights the key concepts of reflective practice and life-long learning, and creates a more inclusive environment [86].

### We must educate respectfully and with inclusion

Culturally capable and safe health professionals are required for improved health care experiences and equitable health care outcomes for Aboriginal and Torres Strait Islander Peoples [29]. Higher education institutions need to include First Nations health curricula to ensure learning outcomes and accreditation requirements are realised, graduating entry-to-practice level podiatrists with the capacity to provide and progress culturally capable and safe care to Aboriginal and Torres Strait Islander Peoples [29, 74, 87].

With overwhelming evidence of the potential of Aboriginal and Torres Strait Islander health curricula to benefit Aboriginal and Torres Strait Islander health outcome equity [29, 74, 87–92] and Bodkin-Andrews & Carlson (2016) indicating ‘the need for the continual acceptance, respect, and promotion of Indigenous voices and identities within the educational environment’ [1], there is growing impetus for curriculum designers to reflect on how this can be achieved in an effective, respectful and inclusive manner.

The Aboriginal and Torres Strait Islander Health Curriculum Framework [29], developed with direction from Aboriginal and Torres Strait Islander representative stakeholder organisations [29], seeks to develop and instil a culturally safe health workforce. The Aboriginal and Torres Strait Islander Health Curriculum Framework describes a Graduate Cultural Capability model for higher education providers and their clinical placement providers, inclusive of the five interconnected cultural capabilities; Respect, Communication, Safety and Quality; Reflection; and Advocacy [29], and, aligning with seventeen learning outcomes derived from Bloom’s revised teaching taxonomy [29, 93]. Placing Aboriginal and Torres Strait Islander Peoples at the centre of health care delivery, the framework describes implementation and accreditation guidelines, directing gold standard and consistent curricula to enable better health outcomes for Aboriginal and Torres Strait Islander Peoples accessing and utilising what they judge to be culturally safe health care [29].

To maintain fundamental change that empowers Aboriginal and Torres Strait Islander Peoples with significant control of the management of their health, the Aboriginal and Torres Strait Islander Health Curriculum Framework needs to be embedded using principles that develop understanding of the cultural dimensions of the health and well-being of Aboriginal and Torres Strait Islander Peoples [94] and that facilitate humble and inclusive teaching and learning. Having Aboriginal and Torres Strait Islander voices and perspectives shape undergraduate podiatry education and clinical placement is an innovative and necessary approach to teaching First Nations health and is promoted in the Aboriginal and Torres Strait Islander Health and Cultural Safety Strategy 2020–2025 [9]. To improve the cultural safety of podiatry in Australia, it is also our responsibility to become safer places of education for more Aboriginal and Torres Strait Islander students to enrol into, and to provide culturally safe podiatry programs that graduate more Aboriginal and Torres Strait Islander practitioners in the future. This fundamental need will be addressed in our future publications.

### We must research respectfully and with reciprocity

The Aboriginal and Torres Strait Islander Health and Cultural Safety Strategy 2020–2025 is central to improving curricula leading to podiatrist registration [9] and by extension, guiding culturally safe research underpinning education. A Western research perspective based upon Eurocentric worldview [1] founds our knowledge of Aboriginal and Torres Strait Islander Peoples and culture through an anthropological lens. It acquires knowledge about people and culture rather than developing knowledge with and from First Nations Peoples. Such overwhelmingly biased research perspective homogenises Aboriginal and Torres Strait Islander identity and worldview [1, 17], is dismissive of Aboriginal and Torres Strait Islander research and its methodologies, and maintains undertones of scientific racism [1]. Colonising Western researchers’ perspectives of Aboriginal and Torres Strait Islander Peoples and culture documented their research subjects as specimens and defined First Nations Peoples as less intelligent whilst using biased, ethnocentric methods that produced invalid results and scarring racial stereotypes [95]. With a powerful and privileged outlook [95] and predicated on racial separatism, scientific racism of the late nineteenth century [52] continues to give Aboriginal and Torres Strait Islander Peoples good reason to remain vigilant of research and researchers alike today and into the future [95].

Use of tools facilitating *good* Aboriginal and Torres Strait Islander research [95–97] and recognition of tens of thousands of years of knowledge delivering prosperous health and well-being to Aboriginal and Torres Strait Islander Peoples prior to colonisation [98] ensures Aboriginal and Torres Strait Islander community consultation and involvement in research. This works to advance Aboriginal and Torres Strait Islander health by use of mutually beneficial methods, and investment in reporting results to, and making a positive difference for, Aboriginal and Torres Strait Islander Peoples [95]. It is an entanglement of different theories of knowledge, mutual acceptance, and trust combining in a shared space that will produce research perspective pursuant of, and contributing to, *good* Aboriginal and Torres Strait Islander health research [95] and culturally safe learning [99]. To this end, the Lowitja Institute [98] and Australian Institute of Aboriginal and Torres Strait Islander Studies (AIATSIS) mandate culturally safe research practices inclusive of Aboriginal and Torres Strait Islander researchers, and lead governance of Aboriginal and Torres Strait Islander research with the highest ethical standards [100]. The Australian Institute of Aboriginal and Torres Strait Islander Studies Act (1989) federal legislation [101], delegates leadership in Aboriginal and Torres Strait Islander research ethics and protocols to AIATSIS [100]. This governance, representing the highest standards in Aboriginal and Torres Strait Islander studies, is expected in all institutions, and is delivered through the Code of Ethics for Aboriginal and Torres Strait Islander Research [102]. Adherence to the code advances First Nations health by ensuring research has a positive impact for Aboriginal and Torres Strait Islander Peoples [103]. The code is underpinned by the four principles of: Indigenous self-determination, Indigenous leadership, Impact and value and, Sustainability and accountability [103].

As a mechanism for delivering ever-developing culturally capable and safe podiatry practice, research underpinning praxis and research involving First Nations Peoples, communities, history and culture, must be conducted within the Code of Ethics for Aboriginal and Torres Strait Islander Research. Our podiatry research must always look to advance First Nations health outcomes and demonstrate respect for and reciprocity with Aboriginal and Torres Strait Islander Peoples.

### We must abide by the United Nations Declaration on the Rights of Indigenous Peoples

In 2007, Australia was one of only four nations to oppose the United Nations Declaration on the Rights of Indigenous Peoples [47, 104]. Today, since reversing that stance, Australia now supports the Declaration, along with over 140 other countries [47, 105, 106].

The United Nations Declaration on the Rights of Indigenous Peoples is a mechanism enabling cultural safety. Articles 2, 3, 4 and 24 of the declaration state that Indigenous Peoples have; ‘the right to be free from any kind of discrimination;’ ‘the right to self-determination;’ ‘the right to autonomy’ [107]; ‘the right to their traditional medicines and to maintain their health practices;’ ‘the right to access, without any discrimination, to all social and health services;’ and ‘an equal right to the enjoyment of the highest attainable standard of physical and mental health’ [107].

The governance developing the Aboriginal and Torres Strait Islander Health and Cultural Safety Strategy 2020–2025 is a joint decision-making body inclusive of independent Aboriginal and Torres Strait Islander health experts, leaders and peak bodies [9]. It aligns intentions and purpose of the strategy with that of the United Nations Declaration on the Rights of Indigenous Peoples [9]. To abide by, and to uphold, the United Nations Declaration on the Rights of Indigenous Peoples, healthcare organisations and individual practitioners need to instil ever culturally safer practice. Enabling self-determination, autonomy and culturally safe health care provision is critical to improving Aboriginal and Torres Strait Islander health outcomes, actualising achieving full realisation of human rights.

## Conclusion

Aboriginal and Torres Strait Islander access to healthcare is reduced by lack of culturally safe service [108], preventing equity in healthcare outcomes in Australia. As podiatrists, we must be part of the solution to this inexcusable shame and begin a journey to improve our culturally capable and safe practice. In communities where provision of care is culturally safe, Aboriginal and Torres Strait Islander Peoples access health services more frequently and have better health outcomes [109]. As podiatrists across all sectors of our profession, revision of truthful Australian history will begin a life-long process developing provision of culturally capable and safe health care; health care inclusive of self-reflexive, non-judgmental and respectful learning, research and practice; health care that engages in power-sharing, communication and mutually beneficial relationships; health care that is free from racism and prejudicial implicit bias. Health care that is acknowledging of the pride, strength, dignity and rights of Aboriginal and Torres Strait Islander Peoples. To achieve ever-developing culturally safe podiatry care, we need to decolonise our podiatry practices and the organisations, systems and settings within which we work, across all First Nations [110].

## Data Availability

The datasets used and/or analysed during the current study are available from the corresponding author upon reasonable request.

## Abbreviations

AHPRA: Australian Health Practitioner Regulation Authority
AIATSIS: Australian Institute of Aboriginal and Torres Strait Islander Studies
